# Interconnected influences of diet, gut microbiome, and metabolome on cognition across three metabolomics platforms

**DOI:** 10.21203/rs.3.rs-9917711/v1

**Published:** 2026-06-11

**Authors:** Lora Khatib, Lucas Patel, Siamak MahmoudianDehkordi, Jennifer S. Labus, Julius Agongo, Kamil Borkowski, Madison Ambre, Christopher Brydges, Leyla Schimmel, Colette Blach, Alzheimer Gut Microbiome Project Consortium, Naama Karu, Matthew Taylor, Edgar Diaz, Jared Brosch, Barbara Bendlin, Russell Swerdlow, Victor W. Henderson, Doris Suyu Chen, Andrew J. Saykin, Suzanne Craft, James Brewer, Thomas Wisniewski, Erik D. Roberson, Pieter C. Dorrestein, Rima Kaddurah-Daouk, Rob Knight

**Affiliations:** 1 Department of Pediatrics, University of California, San Diego, La Jolla, California, USA; 2 Neurosciences Graduate Program, University of California, San Diego, La Jolla, California, USA; 3 Bioinformatics and Systems Biology Program, University of California, San Diego, La Jolla, CA, USA; 4 Medical Scientist Training Program, University of California, San Diego, La Jolla, CA, USA; 5 Department of Psychiatry and Behavioral Sciences, Duke University, Durham, NC, USA; 6 G. Oppenheimer Center for Neurobiology of Stress and Resilience, University of California, Los Angeles, California, USA; 7 Vatche and Tamar Manoukian Division of Digestive Diseases, University of California, Los Angeles, California, USA; 8 UCLA Goodman-Luskin Microbiome Center, University of California, Los Angeles, Integrative Biostatistics and Bioinformatics Core and Neuroimaging Core, California, USA; 9 David Geffen School of Medicine, University of California, Los Angeles, California, USA; 10 Skaggs School of Pharmacy and Pharmaceutical Sciences, University of California, San Diego, La Jolla, CA, USA; 11 Genome Center, University of California, Davis, Davis, CA 95616, USA; 12 Duke Molecular Physiology Institute, Duke University, Durham, NC, 27701, USA; 13 Tasmanian Independent Metabolomics and Analytical Chemistry Solutions (TIMACS), Hobart, Tasmania, 7008, Australia; 14 Department of Dietetics and Nutrition, University of Kansas Medical Center, Kansas City, Kansas 66160; 15 Department of Research and Health Promotion, San Ysidro Health, San Diego, CA; 16 Department of Neurology, Indiana University School of Medicine, Indianapolis, IN; 17 Wisconsin Alzheimer’s Disease Research Center, University of Wisconsin, Madison, WI; 18 Institute of Neuroscience and Physiology, the Sahlgrenska Academy at the University of Gothenburg, Mölndal, Sweden; 19 University of Kansas Alzheimer’s Disease Research Center, Fairway, Kansas 66205; 20 Departments of Epidemiology & Population Health and of Neurology & Neurological Sciences, Stanford University, Stanford, CA 94305; 21 University of California Davis Alzheimer Disease Research Center, Sacramento, CA; 22 Indiana Alzheimer’s Disease Research Center and Department of Radiology and Imaging Sciences, Indiana University School of Medicine, Indianapolis, IN; 23 Wake Forest University School of Medicine Alzheimer’s Disease Research Center, Winston-Salem, NC, 27157; 24 Shiley-Marcos Alzheimer’s Disease Research Center, Department of Neurosciences, University of California, San Diego, La Jolla, CA, USA; 25 New York University Grossman School of Medicine, New York, NY, USA; 26 Department of Neurology, University of Alabama at Birmingham, Birmingham, AL, USA; 27 Center for Microbiome Innovation, University of California, San Diego, La Jolla, CA, USA; 28 Department of Pharmacology, University of California, San Diego, La Jolla, CA, 92093, USA; 29 Collaborative Mass Spectrometry Innovation Center, Skaggs School of Pharmacy and Pharmaceutical Sciences, University of California, San Diego, La Jolla, CA, USA; 30 Duke Institute of Brain Sciences, Duke University, Durham, NC, USA; 31 Department of Medicine, Duke University, Durham, NC, USA; 32 Department of Computer Science and Engineering, University of California, San Diego, La Jolla, CA, USA; 33 Shu Chien-Gene Lay Department of Bioengineering, University of California, San Diego, La Jolla, CA, USA; 34 Halıcıoğlu Data Science Institute, University of California, San Diego, La Jolla, CA, USA; 35 Hong Kong University of Science and Technology Jockey Club Institute for Advanced Study, Hong Kong University of Science and Technology, Hong Kong SAR, China

## Abstract

Cognitive impairment is increasing with global aging, yet mechanisms linking diet, the gut microbiome, and metabolism to cognitive function remain unclear. To investigate a diet-microbiome-metabolome axis associated with cognition, we integrated fecal metagenomics, diet, and multi-platform plasma metabolomics in 505 older adults from four ADRCs. Several microbes broadly associated with circulating metabolites were also linked to multiple measures of cognitive performance. These taxa exhibited coordinated metabolic signatures, with cognition-positive microbes associated with antioxidant, lipid, and microbial–host co-metabolites, and microbes negatively associated with cognition were linked to inflammatory and aromatic amino acid–derived metabolites. Dietary patterns, particularly the Healthy Eating Index Greens and Beans component, were associated with microbial composition and metabolomic structure. Mediation analyses supported a diet–microbe–metabolite–cognition pathway, while metabolites remained associated with cognition after accounting for microbial features. These findings highlight the metabolome as a central integrator of diet, microbial activity, and cognitive function.

With increasing longevity worldwide, cognitive impairment and neurodegenerative diseases pose a growing global health burden.^1^ Although classic neuropathological hallmarks, such as amyloid-β deposition and tau pathology, remain central to Alzheimer’s disease (AD) and related disorders,^2^ there is growing recognition that brain health is shaped by bidirectional communication between the central nervous system and peripheral physiological systems.^3,4^ This concept is encapsulated in the gut–brain axis, a complex network linking the brain with the gastrointestinal tract through neural, immune, endocrine, and metabolic pathways. Through this axis, systemic processes, including metabolism, inflammation, and diet, can influence cognitive function and neurodegenerative disease progression. Understanding how these interconnected systems contribute to brain health is critical for identifying modifiable pathways that may promote cognitive resilience.

A key mediator of the gut–brain axis is the gut microbiome, which plays a central role in regulating host metabolism and immune function.^5,6^ Observational studies have reported altered gut microbiome composition in individuals with AD and mild cognitive impairment, suggesting a potential link between microbial dysbiosis and cognitive decline.^7–9^ Beyond taxonomic shifts, the gut microbiome contributes to the production of a diverse array of metabolites that can enter systemic circulation and influence host physiology.

Recent large-scale multi-omic efforts, including work from our consortium, have identified robust metabolic signatures associated with cognitive function and impairment.^10–15^ Notably, many of these metabolites appear to be influenced by both dietary exposures and the gut microbiome, reflecting complex host–microbe co-metabolic processes. These findings highlight that circulating metabolites often represent integrated outputs of diet, microbial metabolism, and host physiology, rather than isolated signals from a single source.

Despite these connections, relatively few studies have integrated microbiome and metabolomic data to characterize coordinated microbial-metabolic signatures associated with cognitive performance. Moreover, microbial metabolites span a wide range of chemical classes, many of which are not captured by a single analytical platform.^16–18^ Specialized platforms can preferentially detect distinct classes of metabolites such as lipids, amino acid derivatives, and xenobiotics. As a result, integration of multiple metabolomic platforms could help achieve broader coverage of host and microbially derived metabolites.

Diet represents another key upstream factor influencing both the microbiome and host metabolism. However, a major limitation of diet–microbiome studies is the reliance on self-reported dietary data, which is subject to recall bias, measurement error, and cross-cohort inconsistencies.^19^ As a result, associations between diet and downstream biological processes may be attenuated or obscured. Emerging approaches using metabolomic-derived food exposure markers offer a complementary strategy to objectively capture dietary intake and its biochemical consequences.^20^ Recent work has demonstrated that panels of circulating metabolites can serve as robust indicators of specific food groups and dietary patterns, improving the resolution of diet–health associations beyond traditional self-report measures. These metabolite-based proxies reflect both dietary intake and subsequent host and microbial metabolism, providing a more integrated and biologically relevant measure of dietary exposure.

Because diet acts as a complex exposure composed of interacting foods and nutrients, dietary pattern-level measures may better capture biologically meaningful relationships with the microbiome and metabolome than isolated dietary components.^21^ Plant-rich dietary patterns, including those characterized by the Healthy Eating Index (HEI),^22^ have been associated with improved cognitive outcomes and reduced risk of dementia.^23–25^ Diet can also shape microbial community composition and metabolic activity, with higher intake of fiber-rich foods promoting the production of metabolites linked to anti-inflammatory and antioxidant pathways.^26,27^ However, the extent to which diet influences cognition through microbiome-mediated metabolic pathways, and whether metabolomic measures can enhance detection of these relationships, remains largely unresolved.

Here, we leveraged samples acquired by the Alzheimer’s Gut Microbiome Project from six Alzheimer’s disease research centers (ADRCs) to investigate the relationships between reported diet, the gut microbiome, circulating metabolites, and cognitive performance in a large cohort of older adults. We integrated metabolomic measurements across three complementary platforms, enabling more sensitive and broad coverage of host and microbially derived metabolites than is typically achieved with single-platform approaches. In addition to self-reported dietary data, we incorporated metabolomic features that capture diet-related exposures, allowing us to assess dietary influences through both reported intake and biochemical proxies. Using this integrated multi-omics framework, we first identified microbial taxa broadly associated with circulating metabolites across platforms. We then examined whether these metabolite-associated microbes were linked to cognitive performance. Next, we characterized microbial-metabolite interaction patterns associated with cognition and evaluated the role of diet. Finally, we tested whether microbial and metabolomic features mediated associations between diet and cognitive performance. Together, these analyses reveal a coordinated diet-microbiome-metabolome axis associated with cognitive function and highlight potential metabolic pathways through which diet and microbial activity may influence brain health.

## Results

### Participant demographics

Participant characteristics by clinical diagnosis are presented in Table 1. Participants ranged in age from 49 to 98 years, with a mean age of 72 ± 7 years. There were no significant differences between the cognitively impaired (CI; defined as participants with a research diagnosis of MCI or AD dementia) and unimpaired (CU) participants in BMI, sex, race, ethnicity, depression, antidepressant use, p-tau217, amyloid beta 42 or 40 levels, or overall diet quality (HEI). However, mean age differed slightly between groups (Kruskall-Wallis: 6.5, p = 0.04), with cognitively impaired (CI) individuals being, on average, two years older than cognitively unimpaired (CU) participants. As expected, MoCA scores also differed significantly between groups (p < 0.0001). Anxiety levels (p = .04) and p-tau181 concentrations (p < 0.0001) were significantly higher in CI compared to CU individuals.

### Microbial taxa associated with metabolites are linked to cognitive performance and depleted in cognitively impaired individuals.

**Figure 1.**
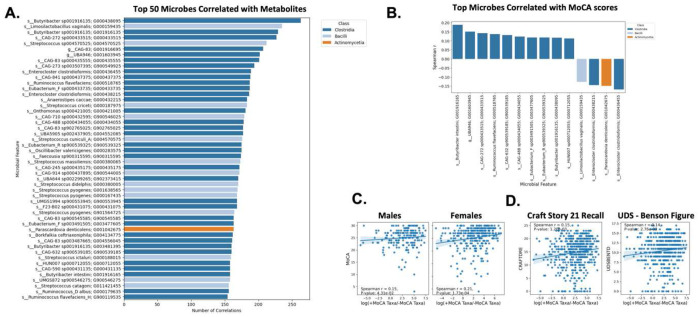
Microbial taxa broadly associated with circulating metabolites are linked to cognitive performance and depleted in cognitively impaired individuals. (A) Top 50 microbial taxa ranked by the number of significant correlations with metabolites across all metabolomic platforms. Bar length indicates the total number of significant associations per taxon (Spearman correlation, FDR-corrected), and colors denote taxonomic class. (B) Subset of metabolite-associated taxa that were also significantly correlated with cognitive performance (MoCA scores). Bar direction indicates the direction of the Spearman correlation with MoCA. (C) Scatterplots illustrating the relationship between MoCA scores and log-ratios derived from the 14 cognition-associated taxa, stratified by sex (males, left; females, right). Each point represents an individual participant. Regression lines with 95% confidence intervals are shown, and Spearman correlation statistics are reported within each panel. (D) Scatterplots showing the relationship between log-ratios derived from the 14 cognition-associated taxa and performance on two cognitive measures: Craft Story 21 Recall (left) and Unified Data Set Benson Figure Delayed Recall (right). Each point represents an individual participant. Regression lines with 95% confidence intervals are shown, and Spearman correlation statistics are reported within each panel.

Across all metabolomic platforms, several microbial taxa showed widespread associations with circulating metabolites (Figure 1A). Many of the top-ranked microbes were correlated with more than 150 metabolites after FDR-correction, indicating broad metabolic connectivity across platforms (and/or potentially shared covariance structure among metabolites across platforms). These associations were observed across three taxonomic classes, with a predominance of taxa from the class *Clostridia* and *Bacilli*. Among the top 50 metabolite-associated microbes, 14 taxa were also significantly correlated with cognitive performance (Figure 1B).

To assess whether the observed microbiome–cognition associations were robust to sex differences, we stratified participants by sex and evaluated the relationship between MoCA scores and a log-ratio derived from the 20 cognition-associated taxa (Figure 1C). In males, the association was modest but significant (Spearman r = 0.15, p = 4.31 × 10^−2^), while in females, the relationship was slightly stronger (r = 0.21, p = 1.73 × 10^−4^). Despite differences in effect size, the directionality of the association was consistent across sexes, supporting the robustness of this microbial signal. Together, these findings suggest that microbial taxa with broad metabolic associations are also linked to cognitive status.

To further evaluate robustness across cognitive domains, we tested this MoCA-associated logratio against additional measures of cognitive performance, including Craft Story 21 Delayed Recall (CRAFTDRE) and Unified Data Set Benson Figure Delayed Recall (UDSBENTD) (Figure 1D). The log-ratio was significantly associated with both CRAFTDRE (r = 0.15, p = 1.20 − 10^−3^) and UDSBENTD (r = 0.14, p = 2.76 × 10^−3^), indicating that this microbial signal is consistently related to cognitive performance across multiple assessments.

### Microbe-metabolite associations cluster by direction of cognitive association.

**Figure 2.**
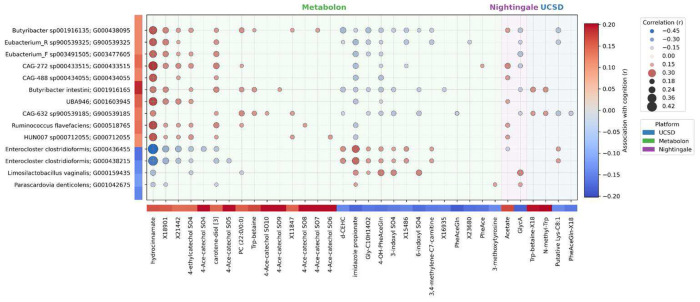
Microbe-metabolite associations cluster by direction of cognitive association. Bubble heatmap showing significant associations between gut microbial species (rows) and circulating metabolites (columns) measured across three metabolomics platforms: Metabolon, Nightingale, and UCSD. Each point represents a statistically significant microbe–metabolite association identified in Spearman r models. Bubble color indicates the direction of the association (positive associations in dark purple; negative associations in pink), while bubble size reflects the strength of the association (absolute effect size). Metabolites are grouped by measurement platform, and microbial taxa are labeled at the species level. Microbe and metabolite names are colored by their relationship with cognition: red text indicates positive associations with cognitive performance, whereas blue text indicates negative associations with cognition. Only associations passing multiple-testing correction are shown.

We identified several significant associations between cognition-associated microbial features and circulating metabolites measured across the three metabolomics platforms (Figure 2). Clear patterns emerged, with microbial taxa exhibiting coordinated relationships with metabolite groups that were either positively or negatively associated with cognitive performance.

For example, among taxa positively associated with cognition, we observed positive associations with metabolites that were also positively associated with cognition and linked to metabolic and antioxidant profiles. Specifically, these taxa were positively correlated with carotenoids (e.g., carotene diol), dietary microbial-human polyphenol co-metabolites (e.g., acetylcatechol sulfate, ethylcatechol sulfate), long-chain phospholipid species (e.g., X1-behenoyl-GPC]), and other dietary indole alkaloids (e.g., tryptophan betaine). Notably, the short-chain fatty acid acetate also demonstrated associations consistent with the cognition-positive microbial cluster.

In contrast, these cognition-positive taxa were inversely associated with several amino acid–derived metabolites arising from host–microbial co-metabolism, including phenylacetylglutamine (derived from phenylalanine), indoxyl sulfate (3-hydroxyindole sulfate) and 6-hydroxyindole sulfate (derived from tryptophan), and imidazole propionate (derived from histidine).

Conversely, taxa negatively associated with cognition, including *Enterocloster clostridioformis, Parascardovia denticolens*, and *Limosilactobacillus vaginalis*, exhibited an opposing metabolic signature. These species were positively associated with metabolites that were themselves negatively associated with cognition and inversely associated with metabolites linked to better cognitive outcomes. Notably, strong correlations were observed with hydrocinnamate (derived from phenylalanine), imidazole propionate, delta-CEHC, and phenylacetylglutamine (derived from phenylalanine). These cognition-negative taxa were also associated with glycoprotein acetylation (GlycA), a composite marker of systemic inflammation.^28^

Across platforms, the metabolomic structure was coherent: taxa positively associated with cognition aligned with antioxidant, lipid remodeling, and potentially anti-inflammatory metabolites, whereas taxa negatively associated with cognition aligned with pro-inflammatory amino acid metabolites.

### Diet Components Linked to the Cognition-Associated Microbial Log-Ratio and Metabolomic Structure

**Figure 3.**
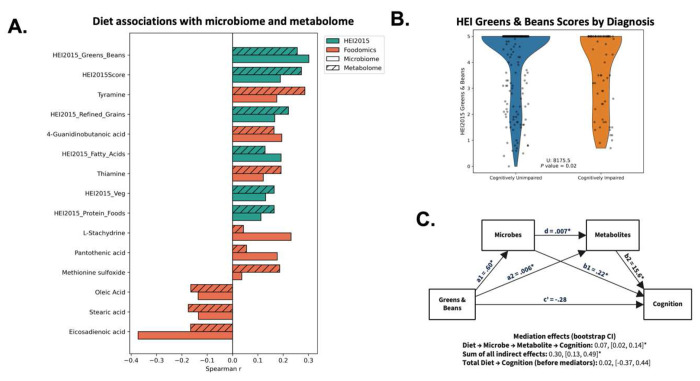
Dietary components linked to cognition-associated microbial and metabolomic signatures. (A) Spearman correlations (x-axis) between individual Healthy Eating Index-2015 (HEI) and Foodomic components (y-axis) and the cognition-associated microbial log-ratio and metabolite PC1 from a joint robust principal component analysis (joint-RPCA) of significant plasma metabolites identified across metabolomic platforms (Figure 2). (**B)** Comparison of HEI Greens & Beans scores between cognitively unimpaired (CU) and cognitively impaired (CI) individuals. Points represent individual participants, and boxplots summarize group distributions. Statistics from a Mann-Whitney U test are shown. **(C)** Conceptual path model and corresponding regression analyses testing whether the HEI-2015 Greens and Beans component is associated with cognitive performance (MoCA) through microbial and metabolomic intermediates. The schematic depicts the serial mediation model.

Healthy Eating Index (HEI-2015) components were constructed using Food Frequency Questionnaires (FFQs) to characterize participants’ dietary patterns. To complement self-reported dietary data, we also incorporated metabolomic-derived food exposure markers (“foodomics”) measured using a validated platform for dietary biomarker detection.^20^ We next sought to identify which dietary components were most strongly associated with cognition-related microbiome and metabolome features. To this end, we performed joint robust principal component analysis (joint-RPCA) on significant plasma metabolites identified across the Metabolon and UCSD platforms (Figure 2). We then tested the association between each dietary component and both the microbial log-ratio and the metabolomic PC1 signature (Figure 3A).

The HEI Greens and Beans component showed the strongest overall associations with both the microbiome (Spearman r = 0.30, q < .0001) and metabolome (r = 0.26, q = .0003), followed by the total HEI score (microbiome: r = 0.19, q = .02; metabolome: r = 0.27, q = .0002) and tyramine (microbiome: r = 0.18, q = .04; metabolome: r = 0.29, q = .0002). In contrast, eicosadienoic acid showed strong negative associations with both the microbiome (r =−0.37, q < .001) and metabolome (r = −0.17, q < .05), along with other fatty acids, including stearic acid and oleic acid. Notably, eicosadienoic acid levels were lower in CI individuals (Mann–Whitney U test, p = .03), suggesting that its relationship with cognition may not be fully captured by its associations with microbiome and metabolome features.

Reported HEI Greens and Beans intake differed between CI and CU participants (Figure 3B; Mann–Whitney U = 8175.5, p = .02). Given its consistent associations with the cognition-associated microbial log-ratio and metabolomic variation, we next tested whether microbial and metabolomic features mediated the relationship between greens and beans intake and cognitive performance (Figure 3C).

In regression models, HEI Greens and Beans was positively associated with the microbial log-ratio (β_a1_ = 0.60, 95% CI = [0.37, 0.80]), indicating that greater intake of greens and beans corresponded to higher abundance of taxa positively associated with cognition. HEI Greens and Beans was also independently associated with the metabolite principal component (β_a2_ = 0.006, 95% CI = [0.001, 0.01]).

The microbial log-ratio was also significantly associated with the metabolite principal component (β_d_ = 0.007, 95% CI = [0.004, 0.01]). In turn, the metabolite principal component was positively associated with MoCA scores (β_b2_ = 15.65, 95% CI = [6.19, 26.46]). The direct association between the microbial log-ratio and cognition was also significant (β_b1_ = 0.22, 95% CI = [0.009, 0.44]).

When modeled jointly, the direct effect of HEI Greens and Beans on cognition was attenuated (c′ = −0.27, 95% CI = [ −0.68, 0.14]), while the total unadjusted effect of diet on cognition prior to inclusion of mediators was small and non-significant (c_total = 0.02, 95% CI = [−0.37, 0.44]).

Bootstrapped mediation analysis revealed a significant serial mediation pathway (Diet → Microbe → Metabolite → Cognition; indirect effect = 0.07, 95% CI = [0.02 to 0.14]). The sum of all indirect effects was significant (0.30, 95% CI = [0.13, 0.49]), indicating that the relationship between greens and beans intake and cognitive performance may operate predominantly through coordinated microbial and metabolomic pathways rather than through a direct effect.

We also evaluated a model in which metabolomic features preceded microbiome variation. This analysis yielded similar total indirect effects, indicating that the overall mediation signal is robust to model specification and suggesting that diet–microbiome–metabolome relationships may be bidirectional.

Together, these findings support a structured diet-microbiome-metabolome axis in which plant-forward dietary intake is associated with cognition through microbial compositional shifts that in turn influence host metabolic profiles linked to cognitive performance.

## Discussion

In this study, we integrated microbiome, multi-platform metabolomic, dietary, and cognitive data to investigate systemic pathways associated with cognitive performance in older adults. Our findings support a coordinated diet–microbiome–metabolome system linked to cognition. Specifically, we identified microbial taxa with broad metabolic connectivity that were associated with cognitive performance and depleted in cognitively impaired individuals, demonstrated that cognition-associated microbes exhibit coherent and opposing metabolic signatures, and showed that plant-forward dietary pattern, particularly the Healthy Eating Index-2015 (HEI) Greens and Beans component, was linked to both microbial composition and metabolomic structure. Mediation analyses are consistent with a pathway in which diet is associated with cognition through coordinated microbial and metabolic pathways rather than through a direct effect.

### Microbial taxa with broad metabolic connectivity are linked to cognition

In the present study, microbial taxa exhibiting widespread associations with circulating metabolites were also linked to cognitive performance. Many of these taxa belonged to the class *Clostridia*, which are known to play key roles in fermentation and metabolic transformation of dietary substrates and have previously been reported to be depleted in AD.^8,29^ The observation that individual taxa were associated with hundreds of metabolites across platforms suggests that certain microbes may be broadly connected to host metabolic variation.

Importantly, taxa positively associated with cognition were relatively depleted in cognitively impaired individuals, suggesting that cognitive impairment is accompanied by shifts in microbial communities that may reduce the production of metabolites linked to beneficial host functions. Prior studies have similarly reported alterations in the gut microbiome in AD and mild cognitive impairment compared to healthy controls,^7,8^ and several of the taxa identified here are consistent with these findings. For example, *Ruminococcus flavefacians* and *Ruminococcus albus* have been associated with greater volume in brain regions that are typically reduced in individuals with AD.^30^ Additionally, *Butrybacter intestinii* and *Butyribacter sp001916135*, are linked to the production of short-chain fatty acids (SCFAs), particularly butyrate, that have been shown to support memory and cognitive function in both animal models and humans.^31,32^

### Microbe-metabolite associations cluster by direction of cognitive association.

Our results further demonstrate that cognition-associated microbes are embedded within coherent metabolic networks. Taxa positively associated with cognition were linked to metabolites that were also positively associated with cognitive performance and inversely associated with metabolites linked to lower cognitive scores. In contrast, taxa negatively associated with cognition exhibited the opposite pattern. Several microbe–metabolite relationships are particularly noteworthy.

Metabolites positively associated with cognition and showed the most consistent associations with cognition-associated microbes included hydrocinnamate, two unknown metabolites (X.18901 and X.21442), 4-ethylcatechol sulfate, carotene diol, 1-behenoyl-GPC, and tryptophan betaine. Hydrocinnamate is a phenylalanine–derived microbial metabolite with known antioxidant activity.^33^ Derivatives of this compound have also been shown to act as agonists of peroxisome proliferator-activated receptors, with antidiabetic and lipid-lowering effects.^34^ However, its role in cognition and dementia has not yet been explored.

Carotene diol (which may include compounds such as lutein) are dietary antioxidants derived from leafy greens and orange-colored fruits and vegetables. These compounds are associated with reduced oxidative stress and disease risk.^35,36^ In the TILDA cohort of over 4,000 individuals aged 50 and older, plasma levels of carotene diols were positively associated with cognitive performance.^37^

Tryptophan betaine has been identified as a biomarker of adherence to dietary patterns associated with reduced frailty in older adults. Serum levels have also been linked to lower blood pressure in individuals with hypertension following the DASH diet (Dietary Approaches to Stop Hypertension), which emphasizes intake of fruits, vegetables, and low-fat dairy, along with diverse protein sources such as poultry.^38^ Mechanistically, tryptophan betaine exhibits anti-inflammatory effects, including attenuation of LPS-induced endothelial inflammation via the PI3K/Akt/mTOR pathway.^39^ It has also been shown to reduce lipid accumulation and inflammation in cellular models of non-alcoholic fatty liver disease (NAFLD) through modulation of p38/JNK and NF-κB signaling pathways.^40^ Additionally, tryptophan betaine may counteract the activity of indole-3-acetic acid and can be metabolized into indoleacrylate, likely via gut microbial processes, which has been shown to promote intestinal epithelial barrier function and reduce inflammation and oxidative stress.^41,42^

In contrast, several metabolites were negatively associated with cognition and cognition-associated microbes, including delta-CEHC, imidazole propionate, Gly-conjugated-C10H14O2, 4-hydroxyphenylacetylglutamine, indoxyl sulfate, GlycA, and phenylacetylglutamine. Imidazole propionate (IMP), a microbial metabolite of histidine, has been associated with impaired metabolic and neurological function, including pathways linked to neurodegeneration, and has been shown to contribute to insulin resistance.^43,44^

Indoxyl sulfate is a microbial-human co-metabolite from dietary tryptophan, that promotes oxidative stress, inflammation, and endothelial dysfunction.^45,46^ It has been linked to cognitive impairment and depression.^47–49^ Elevated levels of indoxyl sulfate have been observed in the brain cortex of Parkinson’s disease patients with dementia compared to those with mild cognitive impairment or normal cognition,^48^ as well as in the plasma and brain of individuals with Alzheimer’s disease relative to controls.^49^ However, its role in broader aging populations remains less well understood and warrants further investigation.

GlycA is a composite biomarker of systemic inflammation reflecting glycosylated acute-phase proteins and is a well-established predictor of cardiovascular disease, chronic inflammation, and mortality risk.^28^ Finally, phenylacetylglutamine is a metabolite formed through human or microbial conjugation of glutamine with phenylacetic acid (from phenylalanine in dietary protein). Its elevated circulating levels have been linked to increased risk of cardiovascular disease, chronic kidney disease, AD, and mortality.^50–52^

Together, these findings highlight microbial taxa that are associated with metabolite profiles linked to cognitive performance and health outcomes, both in our cohort and in prior studies. However, it remains challenging to disentangle whether these microbes directly contribute to the production of these metabolites, whether they co-vary due to shared ecological or metabolic niches, whether they reflect dietary changes associated with MCI or dementia, or whether unmeasured factors drive these associations. Although we accounted for known confounders, causal inference will require longitudinal and experimental validation. Nonetheless, the observed reciprocal patterns support a model in which microbial communities are linked to cognition through coordinated metabolic programs involving inflammation, oxidative stress, and energy metabolism.

### Diet as an upstream driver of microbial and metabolic variation

Our analyses further identify diet as a key upstream factor shaping the observed microbial and metabolomic patterns. Among HEI components, greens and beans intake showed the strongest positive association with the cognition-associated microbial log-ratio and with metabolomic variation. Greens and beans are rich in dietary fiber, polyphenols, and micronutrients that serve as substrates for microbial fermentation and are known to either comprise or promote the production of anti-inflammatory and antioxidant metabolites^53,54^ as well as inducing epigenetic modification.^55^

These findings are consistent with prior work linking plant-forward dietary patterns to improved cognitive outcomes and reduced dementia risk.^24,25,56,57^ Diets such as the Mediterranean diet and other polyphenol-rich dietary patterns, as well as supplementation with probiotics and/or prebiotics, have been shown to increase short-chain fatty acid (SCFA)–producing gut microbes.^58^ SCFAs, along with a range of dietary fibers and bioactive compounds, support intestinal epithelial barrier integrity.^27,59,60^ In turn, increased intestinal permeability has been linked to systemic inflammation and neurodegenerative processes.^61–63^

Other HEI components, including overall diet quality, refined grains, fatty acids, and protein foods, also demonstrated concordant associations with microbiome and metabolome signatures. In parallel, several food-derived metabolites, such as tyramine, 4-guanidinobutanoic acid, thiamine, and L-stachydrine, showed similar cross-omic associations, supporting the idea that circulating dietary biomarkers capture both intake and downstream metabolic processing.

Interestingly, several fatty acids, including eicosadienoic, oleic, and stearic acid, showed consistent inverse relationships across both microbiome and metabolome layers. Eicosadienoic acid, a relatively rare polyunsaturated fatty acid primarily found in animal tissues,^64^ exhibited the strongest negative association with the microbiome signature. Although less well studied than more common fatty acids, eicosadienoic acid has been shown to differentially modulate the production of pro-inflammatory mediators in macrophages.^64^ Notably, it has also been reported to be reduced in individuals with ulcerative colitis,^65^ suggesting a potential link to inflammatory states.

To our knowledge, eicosadienoic acid has not been previously examined in the context of cognition or the microbiome. In the present study, we observed that eicosadienoic acid was also reduced in CI individuals, despite its inverse association with the cognition-associated microbial signature. This pattern suggests that lipid metabolic pathways linked to cognition may operate partly independently of, and in some cases in opposition to, microbiome-associated mechanisms. Further investigation is warranted to clarify the role of eicosadienoic acid in diet–microbiome–metabolome interactions and cognitive health.

Importantly, the concordance between microbiome and metabolome associations for many dietary variables suggests that these two layers may reflect interconnected but partially independent pathways through which diet influences host physiology. For example, plant-derived dietary components may simultaneously promote the growth of beneficial microbial taxa while also contributing directly to circulating metabolites with known biological activity.

Our mediation analyses provide evidence for a structured pathway linking diet, the microbiome, metabolism, and cognitive performance. Specifically, we observed a significant serial mediation pathway in which greens and beans intake was associated with shifts in microbial composition, which in turn were associated with metabolomic changes linked to cognitive performance. Notably, the metabolite principal component showed a strong association with cognition, whereas the direct association between microbial composition and cognition was not significant when modeled jointly.

These findings suggest that microbial influences on cognition may be mediated primarily through metabolic outputs rather than through microbial composition alone. This aligns with emerging frameworks emphasizing microbial metabolic function as a key determinant of host physiology.^5,66^ Interestingly, the association between the metabolome and cognition remained strong and significant even after accounting for microbial composition. This finding is consistent with the understanding that not all circulating metabolites are microbially derived, and that host metabolism, diet, and other physiological processes also contribute substantially to the circulating metabolite pool.^67^ Together, these findings point towards the metabolome as a central integrative layer linking both microbial and host processes to cognitive outcomes.

### Limitations and future directions

Several limitations should be considered. First, this study is cross-sectional, limiting causal inference. Longitudinal and interventional studies will be necessary to determine whether changes in diet or the microbiome lead to corresponding changes in metabolic profiles and cognitive outcomes. Second, circulating metabolites provide insight into systemic physiology but do not directly reflect metabolite concentrations within the central nervous system. Future studies integrating cerebrospinal fluid, neuroimaging, or brain tissue metabolomics could provide additional mechanistic insight.

Future work should also explore whether targeted dietary interventions or microbiome modulation can shift these microbial-metabolic pathways and improve cognitive outcomes. Additionally, integrating metagenomic functional profiling and strain-level analyses may help identify specific microbial pathways driving the observed metabolic signatures.

## Conclusions

In summary, our study identifies coordinated microbial and metabolomic signatures associated with cognitive performance and demonstrates that plant-forward dietary intake is linked to these patterns. These findings support a model in which diet influences cognition indirectly through microbiome-mediated metabolic pathways that impact inflammation, oxidative stress, and mitochondrial function. By integrating microbiome, metabolomic, and dietary data at scale, this work provides new insight into systemic mechanisms linking diet and brain health and highlights potential targets for interventions aimed at promoting cognitive resilience during aging.

## Online Methods

### Participants

The AGMP ADRC Study is a multi-institution collaborative research initiative to define how interconnected factors, such as the metabolome, exposome, diet, lifestyle, gut microbiome, and AD genotypes influence individual vulnerability and progression along the AD continuum via longitudinal observational studies (https://alzheimergut.org/). Participants included in the ADRC study were recruited into the AGMP through 10 participating ADRCs across the United States (US). These individuals are followed longitudinally and are either at risk individuals with normal cognitive abilities, individuals with mild cognitive impairment, or those who carry a diagnosis of dementia. They undergo standardized cognitive assessments. ADRCs also collect biofluid samples, including blood, for analysis of disease markers. Written consent for study participation was obtained by each ADRC under Institutional Review Board (IRB) review and approval. All study procedures were in accordance with the Declaration of Helsinki and allowed deidentified data to be shared among pre-approved researchers.

Plasma samples of AGMP participants were collected at 8 of the 10 centers. The plasma samples were sub-aliquoted by the National Centralized Repository for Alzheimer’s Disease and Related Dementias (NCRAD) as part of the Alzheimer’s Disease Center Fluid Biomarker (ADCFB) Initiative via its blood collection protocol (https://ncrad.org/assets/docs/resource/ADCFB/ADCFB_MOP_-_4.2025.pdf). The phenotypical metadata for the plasma samples were generated using the Uniform Data Set version 3 procedures (https://naccdata.org/data-collection/forms-documentation/uds-3). Individuals from 6 centers in the AGMP ADRC study with both metagenomics and blood plasma available were included in the current analysis. The demographics summary of ADRC participant data used in the study is presented in Table 1.

### Clinical Assessment

#### Montreal Cognitive Assessment (MoCA):

Cognitive function was indexed by the Montreal Cognitive Assessment (MoCA),^68^ a standardized assessment of the cognitive domains of visual perception, executive functioning, language, attention, memory and orientation, administered using established protocols.^68,69^ Scoring was based on a 30-point scale; the participants with 12 years of education or fewer received 1 additional point on the total MoCA score to adjust for education.^68,69^

#### Uniform Data Set (UDS):

Delayed verbal and visual memory were assessed using measures from the National Alzheimer’s Coordinating Center Uniform Data Set neuropsychological battery.^70^ Delayed verbal memory was assessed using the Craft Story 21 Delayed Recall task (CRAFTDRE), in which participants recalled a previously presented story after an approximately 20-minute delay. The paraphrase-scored delayed recall score was used, with higher values indicating better verbal episodic memory performance. Delayed visual memory was assessed using the Benson Figure Delayed Recall task (UDSBENTD), in which participants reproduced a previously copied complex figure from memory after an approximately 10–15-minute delay. Higher scores indicate better visual episodic memory performance.

#### Diagnosis:

Participant diagnosis of dementia and/or mild cognitive impairment (MCI) versus cognitively unimpaired (CU) was determined by a multidisciplinary consensus diagnostic panel and based on National Institute on Aging–Alzheimer’s Association (NIA-AA) criteria.^71,72^ Dementia and MCI groups were subsequently aggregated to the Cognitively Impaired (CI) group.

### Dietary Assessment

#### Healthy Eating Index:

The Viocare interactive Food Frequency Questionnaire (FFQ) and a lifestyle questionnaire were completed by all participants.^73^ The FFQ provided detailed estimates of macronutrient (e.g., carbohydrates, proteins, fats) and micronutrient (e.g., vitamins, minerals) intake, as well as a breakdown of food categories (e.g., fruits, vegetables, meats). The Healthy Eating Index–2015 (HEI–2015) was calculated for each participant using FFQ scores.

#### Foodomics:

Metabolomics data generated using the UCSD platform (see below) were used to derive food-based exposure markers (“foodomics”). Briefly, this platform employs a targeted liquid chromatography–tandem mass spectrometry (LC–MS/MS) workflow for the absolute quantification of a curated panel of food-derived compounds in human plasma.^20^ The assay includes approximately 200 compounds selected from the literature and validated against a diverse set of food items, enabling quantitative mapping of specific molecules to dietary exposures. This library of 200 food-derived compounds was applied to LC–MS/MS untargeted runs from ADRC samples to annotate potential food-derived compounds. Specific food-derived compounds identified through spectral library matching were detected in the ADRC samples, and their corresponding peak areas were extracted for further analysis.

### Fecal sample collection and metagenomic data sequencing

Fecal samples were collected as described previously.^8^ Samples arrived as a frozen fecal slurry in cryovials containing 95% EtOH or frozen stool in conical containers. 200 μL of fecal slurry or 20 mg of stool were transferred into a 1 mL Matrix Tubes (Thermo Fisher Scientific, Waltham, MA, USA) for genomic DNA (gDNA) extraction as previously described.^74^ Ethanol was added to each sample and aliquots of the supernatant were made for metabolomics processing. The samples were then dried prior to DNA extraction. Nucleic acids were isolated using reagents from the MagMAX Microbiome Ultra Nucleic Acid Isolation Kit (Thermo Fisher Scientific) following a modified high-throughput protocol.^75^ This workflow minimized reaction volumes, eliminated the sample-to-bead plate transfer step, and avoided vortex-based bead beating, thereby reducing opportunities for cross-contamination and sample loss.

Extracted gDNA was quantified using the Quant-iT PicoGreen dsDNA Assay (Invitrogen, Waltham, MA, USA) and normalized to 5 ng in 3.5 μL of nuclease-free water prior to library construction. Sequencing libraries were generated using a miniaturized protocol adapted from the KAPA HyperPlus Library Preparation Kit (Roche, Basel, Switzerland).^76^ Library concentrations were measured by PicoGreen assay, and libraries were pooled at equal volumes. The pooled library underwent PCR cleanup (Qiagen, Hilden, Germany) and size selection targeting 300–700 bp using a Pippin HT system (Sage Science, Beverly, MA, USA). Library fragment size distributions were assessed using an Agilent 4200 TapeStation (Agilent Technologies, Santa Clara, CA, USA) following PCR cleanup and size selection to confirm expected library profiles. The pooled library was initially sequenced on an Illumina iSeq 100 to generate low-depth data for normalization. Sample-specific read counts and library concentrations from this run were used to calculate normalization factors, enabling optimized re-pooling to achieve more uniform sequencing depth across samples for subsequent high-throughput sequencing on a NovaSeq 6000 / X Plus platform.^77^ The normalized pool was then PCR cleaned, size selected (300–700 bp), and quality-controlled again using the TapeStation prior to sequencing at the Institute for Genomic Medicine at the University of California, San Diego.

### Multi-platforms metabolomics data acquisition and preprocessing

Data from three complementary targeted metabolomics platforms were used in this study, including Nightingale Health nuclear magnetic resonance metabolomic platform,^17,78–80^ Metabolon metabolomic platform,^18^ and an untargeted platform from University of California San Diego.

1. Nightingale Health, a targeted NMR metabolomic platform (Nightingale Health Ltd, Helsinki, Finland), measures up to 250 metabolic biomarkers, providing absolute concentration of common lipids, specific lipids in lipoprotein subclasses, and various low-molecular weight metabolites, such as amino acids, ketone bodies, and glycolysis metabolites. An established protocol was used.^17,78–80^ In brief, samples were stored at −80°C and thawed overnight at +4°C prior to analysis. Samples were mixed and centrifuged at 3200xg, +4°C for 3 min. Via an automated 8-channel liquid handler (PerkinElmer), an equal volumes of blood samples and NMR measurement buffer (75 mmol/L Na2HPO4, 0.08% sodium 3-(trimethylsilyl)propionate-2,2,3,3-d4 and 0.04% sodium azide in 80%/20% H2O/D2O, pH 7.4)5 were mixed. The mixtures were then measured using an AVANCE III HD NMR spectrometer (500 MHz, Bruker) coupled with a cooled robotic sample changer (SampleJet) as well as a cryogenically cooled tripleresonance probe (CryoProbe Prodigy TCI), prior to quantification using the Nightingale Health’s advanced proprietary software.

No feature had a pooled coefficient of variation (CV) greater than 30%. 6 features were excluded due to more than 40% missingness. 12 samples were excluded due to high missingness. The remaining biomarkers were log2-transformed. The remaining missing values were imputed using the k-nearest-neighbor (KNN) algorithm. Six outlier samples were removed using the LOF method, and extreme outlying biomarker values were imputed using a KNN algorithm. The final dataset contained 242 features for 505 study samples.

1. Metabolon: In the Metabolon platforms, 1316 metabolites of known identity and multiple biochemical pathways were measured along with 349 measurements with unknown identity, and only the metabolites with known identity were used in this study. The sample preparation and UPLC-MS/MS analyses were performed using an established protocol.^18^ In brief, the samples stored at −80°C were prepared via the automated MicroLab STAR^Æ^ system (Hamilton Company). Recovery standards were applied prior to extraction. Protein precipitation was performed by applying methanol and vigorous shaking using GenoGrinder 2000 (Glen Mills) for 2 min prior to centrifugation. The resulting extract was sub-aliquoted prior to different analyses. The extracts were dried, stored under N2 overnight and reconstituted with the solvents compatible with the corresponding UPLC-MS/MS methods in addition to recovery standard(s) (i.e., surrogates) and internal standard(s) (ISTD), prior to analyses. In this platform, four analyses were performed on a system consisting of a UPLC (Waters ACQUITY) and a mass spectrometer (Thermo Scientific Q-Exactive high resolution/accurate mass spectrometer) equipped with the electrospray ionization mode (ESI) and an Orbitrap mass analyzer. The four analyses include the following UPLC step-ups: 1) a reverse phase (RP)-UPLC-MS/MS with positive ion mode detection for relatively hydrophilic compounds, using a stationary phase of C18 column (Waters UPLC BEH C18, 2.1x100 mm, 1.7 μm) and gradient elution with mobile phases of water and methanol (MeOH) with addition of 0.05% perfluoropentanoic acid (PFPA) and 0.1% formic acid (FA); 2) a reverse phase (RP)-UPLC-MS/MS with positive ion mode detection for relatively hydrophilic compounds, using the same C18 column but different mobile phases consisting of MeOH, acetonitrile (ACN), and water with addition of 0.05% PFPA and 0.01% FA and a gradient with higher organic content; 3) RP-UPLC-MS/MS with negative ion mode detection, with another C18 column as the stationary phase and mobile phases of methanol and water with addition of 6.5 mM ammonium bicarbonate (pH 8); and 4) a hydrophilic interaction liquid chromatography (HILIC)/UPLC-MS/MS with negative ion mode detection with a stationary phase of HILIC column (Waters UPLC BEH Amide, 2.1x150 mm, 1.7 μm) and gradient elution with mobile phases of water and ACN with addition of 10mM ammonium formate (pH 10.8). A dynamic exclusion mode was used in the MS analyses to alternate between MS and data-dependent tandem MS scans, with scan range around 70-1000 m/z. The data was processed using the combination of software developed by Metabolon and a library based on authenticated analytical standards maintained by Metabolon. Metabolites were identified based on the retention time and MS/MS spectrum. For multiple-day analyses, batch corrections were applied to normalize data points using the median values of the runs.

Metabolites with over 25% missing values were filtered out, leaving 522 out of an original 1,655 metabolites. Next, using study pooled samples, we calculated CVs for each metabolites and excluded metabolites with CV > 30% (n=71 metabolites were excluded). Metabolite intensities were log2 transformed. Remaining missing values were imputed using a k-nearest-neighbor-based algorithm. Outlier samples in the data were removed using the local outlier factor method (LOF)^81^ implemented in the R package bigutilsr. To account for remaining irregularly high or low single concentrations, values with absolute abundance above q = abs(qnorm(0.0125/n)), with n representing the number of samples, were set to missing. This formula finds the cutoff for values with less than 2.5% two-tailed probability to originate from the same normal distribution as the rest of the measurement values, after applying a Bonferroni-inspired correction factor (division by sample size). These new missing values were then imputed by another round of the k-nearest-neighbor algorithm. The final feature table contained 1,059 features for 505 study samples.

1. UCSD Untargeted Metabolomics: Serum samples were stored at −80 C and thawed on ice for 30 min before extraction. For each serum sample, five different volumes (25, 50, 100, 150, 200 L) were extracted (single extraction for each volume) together with one lab blank (n = 7 lab blanks in total for seven serum samples). Serums were extracted with methanol (containing 1 M sulfadimethoxine-d6 as internal standard); methanol volumes were four times the volume of the serum samples (100, 200, 400, 600, 800 L for the respective serum volume). Samples were sonicated for 10 min, incubated at −20 C for 20 min, and centrifuged at 2000 rpm for 10 min (at 4 C). Supernatants were transferred to a 96 well plate, dried with centrifuge evaporation, and stored at −80 C until analysis. Samples were redissolved in 50/50 acetonitrile/water (v/v) before instrument analysis. 95% ethanol was used to extract metabolites from the fecal samples. 400 μL of 95% EtOH was added to each sample and incubated overnight at room temperature before they were homogenized for 2 min at 1200 rpm on a SPEX 1600 miniG (SPEX SamplePrep, Metuchan, NJ) then centrifuged for 5 min at 3700 rpm. Supernatant (200 μL) was then collected and stored at −80 °C for later acquisition. The samples were then dried in a Savant^™^ SpeedVac^™^ (Thermo Fisher Scientific, Waltham, MA, USA) at 45 °C for 1 hr prior to shotgun metagenomic sequencing.

Metabolite features were preprocessed prior to downstream statistical analyses. Features with more than 30% missing values were removed from the dataset. Remaining zero values were treated as missing and converted to NA. The data were then log2-transformed to reduce skewness and improve normality. Missing values were imputed using k-nearest neighbors imputation. Following imputation, metabolite abundances were z-score scaled to place features on a comparable scale. Finally, plate effects were adjusted using ComBat^82^ with mean-only adjustment, preserving variance structure while removing plate-associated shifts in mean abundance.

### Metagenomic data processing

After sequencing, files were demultiplexed, converted to per-sample-FASTQs, and quality controlled and human sequence filtered using the Qiita^83^ admin plugin: https://github.com/qiita-spots/qp-knight-lab-processing following the recommendations from Guccione et al.^84^ In summary, adapter trimming was performed using fastp.^85^ Each sample was then filtered against GRCh38.p14 and T2T-CHM13v2.0^86^ via alignment using minimap2.^87^ Reads aligning to either human reference genome were discarded. Next, a pangenome index was created using the reference assemblies from the Human Pangenome Reference Consortium release 1,^88^ and reads with high pseudo-matching lengths to the pangenome were discarded. The resulting per-sample-FASTQs were uploaded into Qiita study ID #15448 (https://qiita.ucsd.edu/study/description/15448) and processed with its default processing workflow. The metagenomic workflow maps paired-end reads using Bowtie2 v2.5.4^89^ against Web of Life release 2^90^ (WoL2) with the SHOGUN parameter set.^91^ A feature table is then constructed using woltka v0.1.7.^92^

Preps were aggregated into a single analysis on Qiita.^83^ Coverage values were calculated using MIcrobiome COVerage (micov)^93^, and features with <1% cumulative coverage were excluded.

### Harmonization across omics layers

Samples were aligned across data layers at the participant level. When multiple samples were available for a given participant, plasma samples collected closest in time to fecal samples were retained for analysis. Across metabolomic data layers, metabolites were mapped to their respective annotations, and unknown metabolites were excluded.

To address sparsity and compositionality in the metagenomics data, zero values were imputed using the multiplicative_replacement function from the skbio.stats.composition module in scikit-bio (version 0.5.7), followed by centered log-ratio (CLR) transformation.

### Statistical Analysis

Statistical comparisons of participant demographics across clinical diagnoses were performed using the Kruskal–Wallis rank-sum test for continuous variables and Pearson’s chi-squared test for categorical variables, as appropriate.

A series of exploratory analyses were conducted to identify microbial taxa associated with metabolite profiles across platforms and with cognitive performance. Metagenomic and metabolomic features were first regressed on age, sex, and study site, and residuals from these models were carried forward for downstream analyses. Spearman rank correlations were then computed between each microbial taxon and each metabolite. Resulting p-values were adjusted for multiple comparisons using the Benjamini–Hochberg (BH) false discovery rate procedure. The top 50 microbial taxa with the highest number of significant metabolite associations were retained and subsequently tested for associations with MoCA scores (also adjusted for the same covariates) using Spearman correlations with BH correction. Metabolites were similarly evaluated for associations with cognition using the same analytical framework.

Microbial log-ratios were then constructed by taking the log of the sum of taxa positively associated with cognition divided by the sum of taxa negatively associated with cognition. Associations between these log-ratios and MoCA scores, stratified by sex, were assessed using Spearman correlation. The same approach was applied to additional cognitive measures, including Craft Story 21 Delayed Recall and Unified Data Set Benson Figure Delayed Recall.

Metabolite abundances across platforms were standardized using the StandardScaler function from sklearn.preprocessing. Metabolites measured by Metabolon and UCSD that were significantly associated with cognition were retained and input into a joint robust principal component analysis (joint-RPCA; rclr_transform_tables=False) to derive a latent metabolomic factor across platforms.

Foodomics-derived features were centered log-ratio (CLR) transformed. These values, along with Healthy Eating Index–2015 (HEI) scores, were adjusted for the same covariates as described above. Associations between microbial log-ratios and dietary scores were then assessed using Spearman correlations with BH correction for multiple testing.

Finally, to test the hypothesis that diet influences cognition through microbial and metabolite-mediated pathways, a serial mediation analysis was performed. The independent variable was diet (HEI Greens and Beans component), the mediators were the cognition-associated microbial log-ratio and the first principal component derived from the joint-RPCA metabolomics analysis, and MoCA score served as the outcome. All models were adjusted for age, sex, and study site, consistent with the analytical framework described above.

## Figures and Tables

**Table 1.** Participant Demographics

Parameter	N	Cognitively Unimpaired	Cognitively Impaired	Statistic
No. of participants	505	366 (72.3%)	139 (27.5%)	
Age	505	71.9 ± 7.1	73.8 ± 8.1	6.5
BMI	481	27.2 ± 5.5	27.2 ± 5.3	0.01
Sex: Female	505	234 (63.9%)	78 (56.1%)	2.29
Race: White	505	294 (80.3%)	106 (76.3%)	0.78
Race: Black or African American	505	65 (17.8%)	30 (21.6%)	0.73
Race: Other	505	7 (1.9%)	3 (2.2%)	0
Ethnicity: Hispanic	505	13 (3.6%)	11 (7.9%)	3.33
MoCA	491	26.8 ± 2.4	20.8 ± 5.2	1.68E+02
Depression	505	20 (5.5%)	13 (9.4%)	1.9
Anxiety	505	12 (3.3%)	13 (9.4%)	6.66
Antidepressants	503	96 (26.3%)	48 (34.8%)	3.01
pTau181	368	3.4 ± 2.1	4.7 ± 2.9	2.78E+01
pTau217	31	0.4 ± 0.2	1.0 ± 1.1	0.89
Abeta40	45	264.4 ± 68.8	270.9 ± 65.9	0.02
Abeta42	45	22.4 ± 5.8	20.4 ± 4.8	0.56
HEI2015Score	276	69.3 ± 9.7	67.0 ± 11.0	2.72

## Data Availability

Samples were provided to NCRAD by ten Alzheimer’s Disease Research Centres, including UC San Diego, UC Davis, Stanford University, Kansas University, Wisconsin University, Indiana University, New York University, Wake Forest University, Cleveland Clinic, Nevada, and the University of Alabama at Birmingham. Clinical data can be requested from the National Alzheimer’s Coordinating Centre (naccdata.org/). Data will be available in the Synapse AD Knowledge Portal. Gut microbiome data are stored and accessible via the University of California, San Diego Qiita platform (qiita.ucsd.edu/).

## Code Availability

Data harmonization and statistical analysis scripts are available at: https://github.com/lkhatib/ADRC_cognition_multiomics

## References

[1] Wang S., Jiang Y., Yang A., Meng F. & Zhang J. The Expanding Burden of Neurodegenerative Diseases: An Unmet Medical and Social Need. Aging and disease 0 (2024) doi:10.14336/AD.2024.1071. PMC12339136 39571158

[2] Jack C. R. NIA-AA Research Framework: Toward a biological definition of Alzheimer’s disease. Alzheimer’s & Dementia 14, 535–562 (2018). 10.1016/j.jalz.2018.02.018 PMC5958625 29653606

[3] Livingston G. Dementia prevention, intervention, and care: 2020 report of the Lancet Commission. The Lancet 396, 413–446 (2020). 10.1016/S0140-6736(20)30367-6 PMC7392084 32738937

[4] Holmes C. & Butchart J. Systemic inflammation and Alzheimer’s disease. Biochemical Society Transactions 39, 898–901 (2011). 21787320 10.1042/BST0390898

[5] Visconti A. Interplay between the human gut microbiome and host metabolism. Nat Commun 10, 4505 (2019). 31582752 10.1038/s41467-019-12476-z PMC6776654

[6] Wastyk H. C. Gut-microbiota-targeted diets modulate human immune status. Cell 184, 4137–4153.e14 (2021). 34256014 10.1016/j.cell.2021.06.019 PMC9020749

[7] Liu P. Altered microbiomes distinguish Alzheimer’s disease from amnestic mild cognitive impairment and health in a Chinese cohort. Brain, Behavior, and Immunity 80, 633–643 (2019). 31063846 10.1016/j.bbi.2019.05.008

[8] Kang J. W. Gut microbiome compositional and functional features associate with Alzheimer’s disease pathology. Alzheimer’s & Dementia 21, e70417 (2025). 10.1002/alz.70417 PMC12221809 40604345

[9] Vogt N. M. Gut microbiome alterations in Alzheimer’s disease. Sci Rep 7, 13537 (2017). 29051531 10.1038/s41598-017-13601-y PMC5648830

[10] Schweickart A. Sphingolipid and ceramide associations with tau pathology vary across diverse ethnoracial groups in postmortem brain tissue. Preprint at 10.1101/2025.11.04.25339489 (2025).

[11] Borkowski K. APOE Genotype Influences on The Brain Metabolome of Aging Mice – Role for Mitochondrial Energetics in Mechanisms of Resilience in APOE2 Genotype. Preprint at 10.1101/2025.02.25.640178 (2025). PMC12403941 40898377

[12] Huynh K. Concordant peripheral lipidome signatures in two large clinical studies of Alzheimer’s disease. Nat Commun 11, 5698 (2020). 33173055 10.1038/s41467-020-19473-7 PMC7655942

[13] MahmoudianDehkordi S. Altered bile acid profile associates with cognitive impairment in Alzheimer’s disease—An emerging role for gut microbiome. Alzheimer’s & Dementia 15, 76–92 (2019). 10.1016/j.jalz.2018.07.217 PMC6487485 30337151

[14] Baloni P. Metabolic Network Analysis Reveals Altered Bile Acid Synthesis and Metabolism in Alzheimer’s Disease. Cell Rep Med 1, 100138 (2020). 33294859 10.1016/j.xcrm.2020.100138 PMC7691449

[15] Karu N. Exposome contribution to the brain metabolome: importance of body brain connection. Preprint at 10.64898/2026.05.05.26352469 (2026).

[16] Zhang L. A Comprehensive LC–MS Metabolomics Assay for Quantitative Analysis of Serum and Plasma. Metabolites 14, 622 (2024). 39590858 10.3390/metabo14110622 PMC11596266

[17] W¸rtz P. Quantitative Serum Nuclear Magnetic Resonance Metabolomics in Large-Scale Epidemiology: A Primer on -Omic Technologies. American Journal of Epidemiology 186, 1084–1096 (2017). 29106475 10.1093/aje/kwx016 PMC5860146

[18] Ford L. Precision of a Clinical Metabolomics Profiling Platform for Use in the Identification of Inborn Errors of Metabolism. The Journal of Applied Laboratory Medicine 5, 342–356 (2020). 32445384 10.1093/jalm/jfz026

[19] Ottaviani J. I., Sagi-Kiss V., Schroeter H. & Kuhnle G. G. Reliance on self-reports and estimated food composition data in nutrition research introduces significant bias that can only be addressed with biomarkers. eLife 13, RP92941 (2024). 38896457 10.7554/eLife.92941 PMC11186626

[20] Agongo J. Quantitative Food Compounds Enable Dietary Ontology Referencing across 500 Foods and Human Plasma. Anal. Chem. 98, 3160–3176 (2026). 41549429 10.1021/acs.analchem.5c06769 PMC12874208

[21] Cotillard A. A posteriori dietary patterns better explain variations of the gut microbiome than individual markers in the American Gut Project. The American Journal of Clinical Nutrition 115, 432–443 (2022). 34617562 10.1093/ajcn/nqab332 PMC8827078

[22] Krebs-Smith S. M. Update of the Healthy Eating Index: HEI-2015. Journal of the Academy of Nutrition and Dietetics 118, 1591–1602 (2018). 30146071 10.1016/j.jand.2018.05.021 PMC6719291

[23] Fan Y. Association between healthy eating index-2015 and various cognitive domains in US adults aged 60 years or older: the National Health and Nutrition Examination Survey (NHANES) 2011–2014. BMC Public Health 21, 1862 (2021). 34654401 10.1186/s12889-021-11914-2 PMC8520277

[24] Devranis P. Mediterranean Diet, Ketogenic Diet or MIND Diet for Aging Populations with Cognitive Decline: A Systematic Review. Life 13, 173 (2023). 36676122 10.3390/life13010173 PMC9866105

[25] Dilmore A. H. Effects of a ketogenic and low-fat diet on the human metabolome, microbiome, and foodome in adults at risk for Alzheimer’s disease. Alzheimer’s & Dementia 19, 4805–4816 (2023). 10.1002/alz.13007 PMC10551050 37017243

[26] David L. A. Diet rapidly and reproducibly alters the human gut microbiome. Nature 505, 559–563 (2014). 24336217 10.1038/nature12820 PMC3957428

[27] Koh A., De Vadder F., Kovatcheva-Datchary P. & Bäckhed F. From Dietary Fiber to Host Physiology: Short-Chain Fatty Acids as Key Bacterial Metabolites. Cell 165, 1332–1345 (2016). 27259147 10.1016/j.cell.2016.05.041

[28] Mehta N. N., Dey A. K., Maddineni R., Kraus W. E. & Huffman K. M. GlycA measured by NMR spectroscopy is associated with disease activity and cardiovascular disease risk in chronic inflammatory diseases. American Journal of Preventive Cardiology 4, 100120 (2020). 34327480 10.1016/j.ajpc.2020.100120 PMC8315361

[29] Lopetuso L. R., Scaldaferri F., Petito V. & Gasbarrini A. Commensal Clostridia: leading players in the maintenance of gut homeostasis. Gut Pathog 5, 23 (2013). 23941657 10.1186/1757-4749-5-23 PMC3751348

[30] Labus J. S. Ruminococcus-based metagenomic-brain signature linked to cognitive performance and Alzheimer’s disease markers. Alzheimer’s & Dementia 20, e089941 (2024).

[31] Wang X., Wang Z., Cao J., Dong Y. & Chen Y. Gut microbiota-derived metabolites mediate the neuroprotective effect of melatonin in cognitive impairment induced by sleep deprivation. Microbiome 11, 17 (2023). 36721179 10.1186/s40168-022-01452-3 PMC9887785

[32] Wei H. Butyrate ameliorates chronic alcoholic central nervous damage by suppressing microglia-mediated neuroinflammation and modulating the microbiome-gut-brain axis. Biomedicine & Pharmacotherapy 160, 114308 (2023). 36709599 10.1016/j.biopha.2023.114308

[33] Roleira F. M. F. Lipophilic phenolic antioxidants: Correlation between antioxidant profile, partition coefficients and redox properties. Bioorganic & Medicinal Chemistry 18, 5816–5825 (2010). 20650639 10.1016/j.bmc.2010.06.090

[34] Raval P. Revisiting glitazars: Thiophene substituted oxazole containing α-ethoxy phenylpropanoic acid derivatives as highly potent PPARα/γ dual agonists devoid of adverse effects in rodents. Bioorganic & Medicinal Chemistry Letters 21, 3103–3109 (2011). 21450468 10.1016/j.bmcl.2011.03.020

[35] Burri B. J., La Frano M. R. & Zhu C. Absorption, metabolism, and functions of β-cryptoxanthin. Nutr Rev 74, 69–82 (2016). 26747887 10.1093/nutrit/nuv064 PMC4892306

[36] Jomova K. & Valko M. Health protective effects of carotenoids and their interactions with other biological antioxidants. European Journal of Medicinal Chemistry 70, 102–110 (2013). 24141200 10.1016/j.ejmech.2013.09.054

[37] Feeney J. Plasma Lutein and Zeaxanthin Are Associated With Better Cognitive Function Across Multiple Domains in a Large Population-Based Sample of Older Adults: Findings from The Irish Longitudinal Study on Aging. J Gerontol A Biol Sci Med Sci 72, 1431–1436 (2017). 28329221 10.1093/gerona/glw330

[38] Kim H. Metabolomic Profiles Associated With Blood Pressure Reduction in Response to the DASH and DASH-Sodium Dietary Interventions. Hypertension 80, 1494–1506 (2023). 37161796 10.1161/HYPERTENSIONAHA.123.20901 PMC10262995

[39] Sun H., Zhu X., Cai W. & Qiu L. Hypaphorine Attenuates Lipopolysaccharide-Induced Endothelial Inflammation via Regulation of TLR4 and PPAR-γ Dependent on PI3K/Akt/mTOR Signal Pathway. IJMS 18, 844 (2017). 28420166 10.3390/ijms18040844 PMC5412428

[40] Yanko R. Tryptophan Prevents the Development of Non-Alcoholic Fatty Liver Disease. DMSO Volume 16, 4195–4204 (2023). 10.2147/DMSO.S444278 PMC10752026 38152280

[41] Kumar P., Lee J. & Lee J. Diverse roles of microbial indole compounds in eukaryotic systems. Biological Reviews 96, 2522–2545 (2021). 34137156 10.1111/brv.12765 PMC9290978

[42] Marella B. A seven-year longitudinal study of the Alzheimer’s disease blood metabolome. medRxiv 2025.12.05.25341709 (2025) doi:10.64898/2025.12.05.25341709.

[43] Vemuganti V. Gut bacterial metabolite imidazole propionate potentiates Alzheimer’s disease pathology. Preprint at 10.1101/2025.06.08.657719 (2025). PMC13454616 42362546

[44] Agirman G. The microbial metabolite imidazole propionate modulates hypothalamic activity and stress-induced behaviors. Cell Host & Microbe 33, 2030–2042.e9 (2025). 41297540 10.1016/j.chom.2025.10.019 PMC12811846

[45] Adesso S. Indoxyl Sulfate Affects Glial Function Increasing Oxidative Stress and Neuroinflammation in Chronic Kidney Disease: Interaction between Astrocytes and Microglia. Front Pharmacol 8, 370 (2017). 28659803 10.3389/fphar.2017.00370 PMC5466960

[46] Dou L. The uremic solute indoxyl sulfate induces oxidative stress in endothelial cells. Journal of Thrombosis and Haemostasis 5, 1302–1308 (2007). 17403109 10.1111/j.1538-7836.2007.02540.x

[47] Brydges C. R. Indoxyl sulfate, a gut microbiome-derived uremic toxin, is associated with psychic anxiety and its functional magnetic resonance imaging-based neurologic signature. Sci Rep 11, 21011 (2021). 34697401 10.1038/s41598-021-99845-1 PMC8546034

[48] Kalecký K. & Bottiglieri T. Targeted metabolomic analysis in Parkinson’s disease brain frontal cortex and putamen with relation to cognitive impairment. NPJ Parkinsons Dis 9, 84 (2023). 37270646 10.1038/s41531-023-00531-y PMC10239505

[49] Kalecký K, German D. C, Montillo A. A. & Bottiglieri T. Targeted Metabolomic Analysis in Alzheimer’s Disease Plasma and Brain Tissue in Non-Hispanic Whites. J Alzheimers Dis 86, 1875–1895 (2022). 35253754 10.3233/JAD-215448 PMC9108583

[50] Krishnamoorthy N. K. Role of the Gut Bacteria-Derived Metabolite Phenylacetylglutamine in Health and Diseases. ACS Omega acsomega.3c08184 (2024) doi:10.1021/acsomega.3c08184. PMC10809373 38284070

[51] González-Domínguez R, Javier Rupérez F, García-Barrera T, Barbas C. & Luis Gómez-Ariza J. Metabolomic-Driven Elucidation of Serum Disturbances Associated with Alzheimer’;s Disease and Mild Cognitive Impairment. CAR 13, 641–653 (2016). 10.2174/1567205013666160129095138 26825096

[52] Liang N. Peripheral inflammation is associated with brain atrophy and cognitive decline linked to mild cognitive impairment and Alzheimer’s disease. Sci Rep 14, 17423 (2024). 39075118 10.1038/s41598-024-67177-5 PMC11286782

[53] Meiners F., Ortega-Matienzo A., Fuellen G. & Barrantes I. Gut microbiome-mediated health effects of fiber and polyphenol-rich dietary interventions. Front. Nutr. 12, 1647740 (2025). 40948860 10.3389/fnut.2025.1647740 PMC12425962

[54] Sejbuk M., Mirończuk-Chodakowska I., Karav S. & Witkowska A. M. Dietary Polyphenols, Food Processing and Gut Microbiome: Recent Findings on Bioavailability, Bioactivity, and Gut Microbiome Interplay. Antioxidants 13, 1220 (2024). 39456473 10.3390/antiox13101220 PMC11505337

[55] Nohesara S., Abdolmaleky H. M. & Thiagalingam S. Epigenetic Aberrations in Major Psychiatric Diseases Related to Diet and Gut Microbiome Alterations. Genes (Basel) 14, 1506 (2023). 37510410 10.3390/genes14071506 PMC10379841

[56] Morris M. C. MIND diet associated with reduced incidence of Alzheimer’s disease. Alzheimer’s & Dementia 11, 1007–1014 (2015). 10.1016/j.jalz.2014.11.009 PMC4532650 25681666

[57] Tu J., Zhang J. & Chen G. Higher dietary butyrate intake is associated with better cognitive function in older adults: evidence from a cross-sectional study. Front. Aging Neurosci. 17, 1522498 (2025). 40224959 10.3389/fnagi.2025.1522498 PMC11985818

[58] Ghosh T. S., Shanahan F. & O’Toole P. W. The gut microbiome as a modulator of healthy ageing. Nat Rev Gastroenterol Hepatol 19, 565–584 (2022). 35468952 10.1038/s41575-022-00605-x PMC9035980

[59] Vernocchi P., Del Chierico F. & Putignani L. Gut Microbiota Profiling: Metabolomics Based Approach to Unravel Compounds Affecting Human Health. Front Microbiol 7, 1144 (2016). 27507964 10.3389/fmicb.2016.01144 PMC4960240

[60] Dalile B., Van Oudenhove L., Vervliet B. & Verbeke K. The role of short-chain fatty acids in microbiota-gut-brain communication. Nat Rev Gastroenterol Hepatol 16, 461–478 (2019). 31123355 10.1038/s41575-019-0157-3

[61] Li Q. The role of the microbiota-gut-brain axis and intestinal microbiome dysregulation in Parkinson’s disease. Front. Neurol. 14, 1185375 (2023). 37305758 10.3389/fneur.2023.1185375 PMC10249504

[62] Mirzaei R. Role of microbiota-derived short-chain fatty acids in nervous system disorders. Biomedicine & Pharmacotherapy 139, 111661 (2021). 34243604 10.1016/j.biopha.2021.111661

[63] Raber J. & Sharpton T. J. Gastrointestinal Dysfunction in Neurological and Neurodegenerative Disorders. Semin Neurol 43, 634–644 (2023). 37607587 10.1055/s-0043-1771459 PMC10953489

[64] Huang Y.-S., Huang W.-C., Li C.-W. & Chuang L.-T. Eicosadienoic acid differentially modulates production of pro-inflammatory modulators in murine macrophages. Mol Cell Biochem 358, 85–94 (2011). 21688154 10.1007/s11010-011-0924-0

[65] Sitkin S. & Pokrotnieks J. Alterations in Polyunsaturated Fatty Acid Metabolism and Reduced Serum Eicosadienoic Acid Level in Ulcerative Colitis: Is There a Place for Metabolomic Fatty Acid Biomarkers in IBD? Dig Dis Sci 63, 2480–2481 (2018). 29987625 10.1007/s10620-018-5182-5

[66] Nicholson J. K. Host-Gut Microbiota Metabolic Interactions. Science 336, 1262–1267 (2012). 22674330 10.1126/science.1223813

[67] Nicholson J. K. Host-Gut Microbiota Metabolic Interactions. Science 336, 1262–1267 (2012). 22674330 10.1126/science.1223813

[68] Nasreddine Z. S. The Montreal Cognitive Assessment, MoCA: A Brief Screening Tool For Mild Cognitive Impairment. J American Geriatrics Society 53, 695–699 (2005). 10.1111/j.1532-5415.2005.53221.x 15817019

[69] Julayanont P. The Montreal Cognitive Assessment—Basic: A Screening Tool for Mild Cognitive Impairment in Illiterate and Low-Educated Elderly Adults. J American Geriatrics Society 63, 2550–2554 (2015). 10.1111/jgs.13820 26648041

[70] Weintraub S. Version 3 of the Alzheimer Disease Centers’ Neuropsychological Test Battery in the Uniform Data Set (UDS). Alzheimer Disease & Associated Disorders 32, 10–17 (2018). 29240561 10.1097/WAD.0000000000000223 PMC5821520

[71] McKhann G. M. The diagnosis of dementia due to Alzheimer’s disease: Recommendations from the National Institute on Aging-Alzheimer’s Association workgroups on diagnostic guidelines for Alzheimer’s disease. Alzheimer’s & Dementia 7, 263–269 (2011). 10.1016/j.jalz.2011.03.005 PMC3312024 21514250

[72] Albert M. S. The diagnosis of mild cognitive impairment due to Alzheimer’s disease: Recommendations from the National Institute on Aging-Alzheimer’s Association workgroups on diagnostic guidelines for Alzheimer’s disease. Alzheimer’s & Dementia 7, 270–279 (2011). 10.1016/j.jalz.2011.03.008 PMC3312027 21514249

[73] Kristal A. R. Evaluation of Web-Based, Self-Administered, Graphical Food Frequency Questionnaire. Journal of the Academy of Nutrition and Dietetics 114, 613–621 (2014). 24462267 10.1016/j.jand.2013.11.017 PMC3966309

[74] Brennan C. Clearing the plate: a strategic approach to mitigate well-to-well contamination in large-scale microbiome studies. mSystems 9, e0098524 (2024). 39283083 10.1128/msystems.00985-24 PMC11494942

[75] Shaffer J. P. A comparison of DNA/RNA extraction protocols for high-throughput sequencing of microbial communities. Biotechniques 70, 149–159 (2021). 33512248 10.2144/btn-2020-0153 PMC7931620

[76] Sanders J. G. Optimizing sequencing protocols for leaderboard metagenomics by combining long and short reads. Genome Biol 20, 226 (2019). 31672156 10.1186/s13059-019-1834-9 PMC6822431

[77] Brennan C. Maximizing the potential of high-throughput next-generation sequencing through precise normalization based on read count distribution. mSystems 8, e0000623 (2023). 37350611 10.1128/msystems.00006-23 PMC10469589

[78] Soininen P., Kangas A. J., W¸rtz P, Suna T. & Ala-Korpela M. Quantitative Serum Nuclear Magnetic Resonance Metabolomics in Cardiovascular Epidemiology and Genetics. Circ Cardiovasc Genet 8, 192–206 (2015). 25691689 10.1161/CIRCGENETICS.114.000216

[79] Soininen P. High-throughput serum NMR metabonomics for cost-effective holistic studies on systemic metabolism. Analyst 134, 1781 (2009). 19684899 10.1039/b910205a

[80] Julkunen H. Atlas of plasma NMR biomarkers for health and disease in 118,461 individuals from the UK Biobank. Nat Commun 14, 604 (2023). 36737450 10.1038/s41467-023-36231-7 PMC9898515

[81] Breunig M. M., Kriegel H.-P., Ng R. T. & Sander J. LOF: identifying density-based local outliers. in Proceedings of the 2000 ACM SIGMOD international conference on Management of data 93–104 (ACM, Dallas Texas USA, 2000). doi:10.1145/342009.335388.

[82] Johnson W. E., Li C. & Rabinovic A. Adjusting batch effects in microarray expression data using empirical Bayes methods. Biostatistics 8, 118–127 (2007). 16632515 10.1093/biostatistics/kxj037

[83] Gonzalez A. Qiita: rapid, web-enabled microbiome meta-analysis. Nat Methods 15, 796–798 (2018). 30275573 10.1038/s41592-018-0141-9 PMC6235622

[84] Knight R. Incomplete human reference genomes can drive false sex biases and expose patient-identifying information in metagenomic data. Preprint at 10.21203/rs.3.rs-4721159/v1 (2024). PMC11742726 39827261

[85] Chen S., Zhou Y., Chen Y. & Gu J. fastp: an ultra-fast all-in-one FASTQ preprocessor. Bioinformatics 34, i884–i890 (2018). 30423086 10.1093/bioinformatics/bty560 PMC6129281

[86] Rhie A. The complete sequence of a human Y chromosome. Nature 621, 344–354 (2023). 37612512 10.1038/s41586-023-06457-y PMC10752217

[87] Li H. Minimap2: pairwise alignment for nucleotide sequences. Bioinformatics 34, 3094–3100 (2018). 29750242 10.1093/bioinformatics/bty191 PMC6137996

[88] Liao W.-W. A draft human pangenome reference. Nature 617, 312–324 (2023). 37165242 10.1038/s41586-023-05896-x PMC10172123

[89] Langmead B. & Salzberg S. L. Fast gapped-read alignment with Bowtie 2. Nat Methods 9, 357–359 (2012). 22388286 10.1038/nmeth.1923 PMC3322381

[90] Zhu Q. Phylogenomics of 10,575 genomes reveals evolutionary proximity between domains Bacteria and Archaea. Nat Commun 10, 5477 (2019). 31792218 10.1038/s41467-019-13443-4 PMC6889312

[91] Hillmann B. Evaluating the Information Content of Shallow Shotgun Metagenomics. mSystems 3, 10.1128/msystems.00069-18 (2018). PMC6234283 30443602

[92] Zhu Q. Phylogeny-Aware Analysis of Metagenome Community Ecology Based on Matched Reference Genomes while Bypassing Taxonomy. mSystems 7, e00167–22 (2022). 35369727 10.1128/msystems.00167-22 PMC9040630

[93] Weng Y. Calculating fast differential genome coverages among metagenomic sources using micov. Commun Biol 8, 1624 (2025). 41266796 10.1038/s42003-025-09007-6 PMC12635244

